# Emission Enhancement and Energy Transfers in YV_0.5_P_0.5_O_4_ Nanoparticles Codoped with Eu^3+^ and Bi^3+^ Ions

**DOI:** 10.1021/acs.inorgchem.2c01465

**Published:** 2022-07-28

**Authors:** Marta Wujczyk, Sara Targonska, Philippe Boutinaud, John M. Reeks, Adam Watras, Rafal J. Wiglusz

**Affiliations:** † Institute of Low Temperature and Structure Research, PAS, Okolna 2, 50-422 Wroclaw, Poland; ‡ Université Clermont Auvergne, Clermont Auvergne INP, CNRS, ICFC, F-63000 Clermont-Ferrand, France

## Abstract

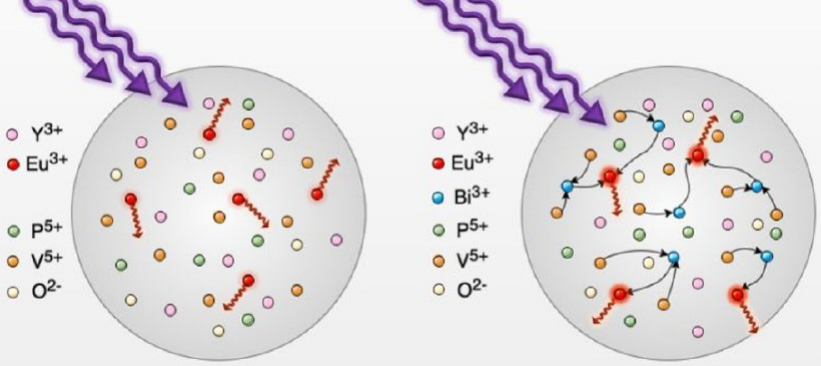

In this study, solid-state solutions of yttrium orthovanadate-phosphate with varying concentrations of codopants (Eu^3+^, Bi^3+^) have been obtained via coprecipitation. An ionic radii mismatch between V^5+^ and P^5+^ substituents is manifested in broad XRD lines. The sharpening of the XRD lines is observed with increasing bismuth ions concentration in the Eu^3+^ codoped YV_0.5_P_0.5_O_4_ matrix. The difference in the number of the Stark components for the ^5^D_0_ → ^7^F_J_ transitions indicates changes in the lattice and a number of possible Eu^3+^ sites. A thorough, systematic spectroscopic analysis of YV_0.5_P_0.5_O_4_: *x* mol % Eu^3+^, *y* mol % Bi^3+^ was conducted at room temperature and 5 K. Metal-to-metal energy transfers occurring between Eu^3+^, V^5+^, and Bi^3+^ optically active ions have been investigated. Additionally, efficiency of the Bi^3+^-Eu^3+^ energy transfer (ET) was calculated.

## Abstract 2

Yttrium orthovanadate-phosphate nanoparticles codoped with varying concentrations of Eu^3+^ and Bi^3+^ ions were synthesized using a wet chemical coprecipitation method. X-ray diffractograms revealed the YV_0.5_P_0.5_O_4_ crystal reordering structure in response to increasing Bi^3+^ concentration. Furthermore, changes in low and room temperature spectroscopic properties were observed. Increases in Bi^3+^ and Eu^3+^ ion concentrations enhanced europium ion emission through MMCT between vanadium, bismuth, and europium ions.

## 1 Introduction

The yttrium orthovanadate and yttrium orthophosphate matrices, doped with europium ions, are popular luminescent phosphors. This is due to their potential applications as laser host materials, polarizers, solar cells, light emitting diodes, host materials for optically active ions, etc.^1−7^

YVO_4_ and YPO_4_ crystallize in the zircon tetragonal system, within the space group *I4*_*1*_*/amd*.^8,9^ Hence, a solid-state solution of yttrium orthovanadate-phosphate can be formed.^10^ Considering YP_0.5_V_0.5_O_4_, its unit cell is composed of 50 mol % vanadium tetrahedral and 50 mol % phosphate tetrahedral groups, statistically substituted. Furthermore, in this work, yttrium ions in the lattice are statistically substituted with europium and bismuth ions.

**Figure 1:**
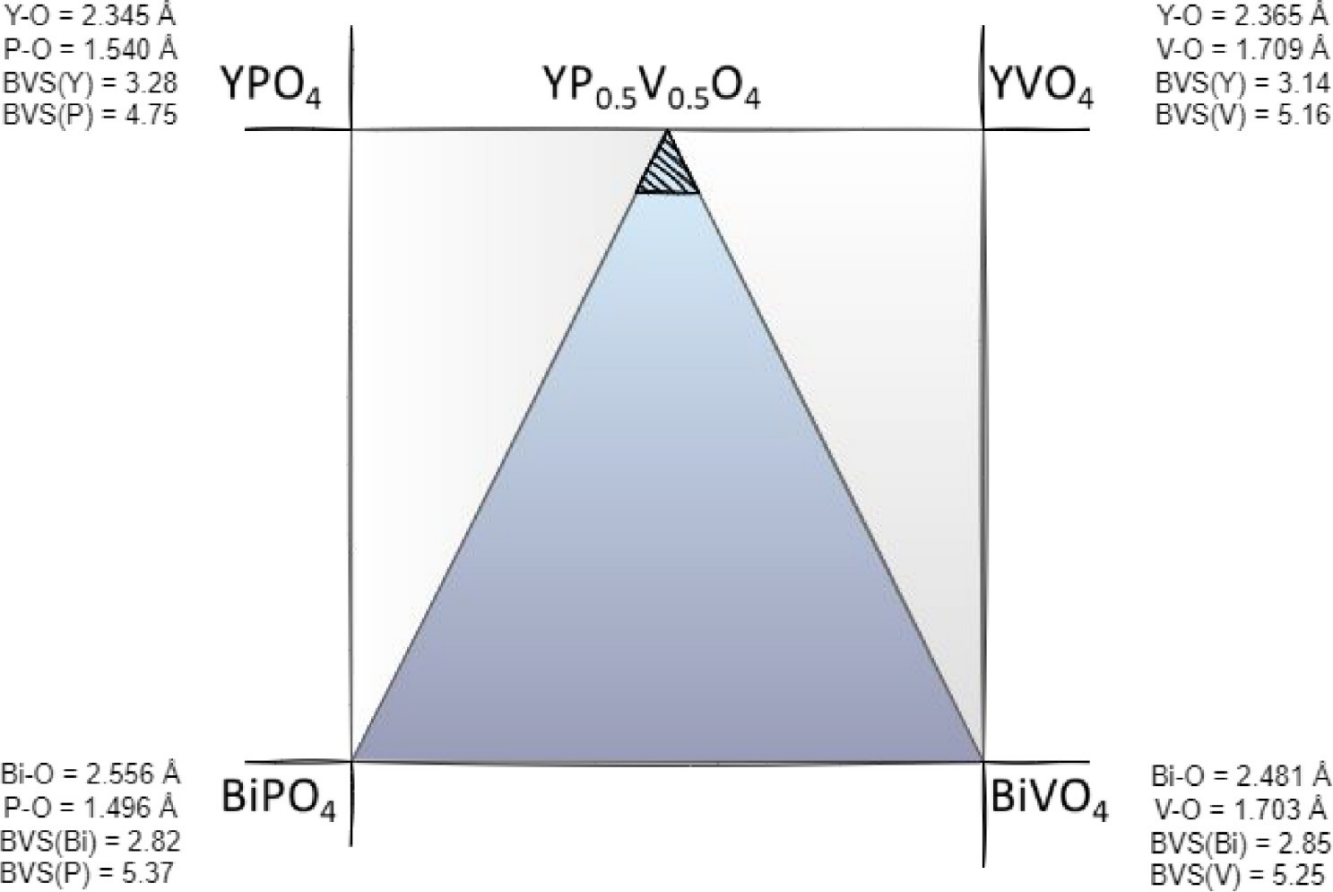
YPO_4_–YVO_4_–BiVO_4_–BiPO_4_ pseudoquaternary diagram. BVS = bond valence sum.

In the present work, the fraction of the YPO_4_–YVO_4_–BiVO_4_–BiPO_4_ pseudoquaternary diagram (more precisely the shaded area of the YP_0.5_V_0.5_O_4_–BiVO_4_–BiPO_4_ pseudo ternary subdiagram) is investigated (Figure 1). The bond valence sums (BVS) were obtained from VESTA,^11^ and bond valence parameters were compiled in ref (12). The compounds in the shaded area have a disordered zircon-like crystal structure due to the inner structural characteristics of YPO_4_ and YVO_4_. Optically active europium ions are incorporated in YP_0.5_V_0.5_O_4_ and (Y,Bi)P_0.5_V_0.5_O_4_ for two purposes: to collect information on the local crystal structure and to investigate energy transfer processes involving Bi^3+^. This paper constitutes an extension of previous reports^2,13−16^ with more systematic, thorough spectroscopic analysis. Previous works^2,13,14^ focus on parameters affecting the Eu^3+^ emission intensity in micron-sized^14^ and nanosized^2,13^ systems. It was previously established that codoping YVO_4_:Eu^3+^ with P^5+^, Bi^3+^, and Gd^3+^ greatly enhances the europium ions’ emission intensity. The compositions which maximize Eu^3+^ emission intensity are typically Y_0.9_Bi_0.05_Eu_0.05_P_0.5_V_0.5_O_4_^13^ or Y_0.45_Gd_0.45_Bi_0.05_Eu_0.05_P_0.5_V_0.5_O_4_.^2^ The reasons for this remain obscure.

Trivalent bismuth ion is known as an attractive activator in zircon vanadates.^15,17−23^ Furthermore, it has been observed that Bi^3+^ is an efficient luminescence sensitizer for trivalent lanthanide ions.^3,24−28^ In this study bismuth and europium ions are chosen as codopants, bismuth ions improve the photoluminescence intensity of Eu^3+^. This phenomenon occurs as a result of UV excitation. This is due to the CT transitions from the Bi^3+^ 6s energy level to the 5d levels of the vanadate and subsequent energy transfer (ET) to Eu^3+^ ions 4f orbitals.^24,29−33^

This work emphasizes characterization of the luminescent properties of these two dopants incorporated into YV_0.5_P_0.5_O_4_. This matrix has a disordered structure as a result of phosphate and vanadate units being randomly dispersed throughout the lattice. This occurs because the vanadate units are ∼8% larger than the phosphates. The extent to which this disorder contributes to the efficiency of Bi^3+^-Eu^3+^ ET will be investigated in this work. To this end, two series of materials were synthesized. The first one was doped with varying amounts of bismuth ions, while the second was doped with varying amounts of europium ions. The chemical compositions involved doping and codoping YV_0.5_P_0.5_O_4_ with *x*Bi^3+^, *y*Eu^3+^ where *x* = 0, 1, 3, 5, 10, 15 mol % and *y* = 0.5, 1, 2, 5 mol %. The solid state solutions were obtained by the wet chemistry synthesis-coprecipitation method with additional heat-treatment at 800 °C for 3 h.

## 2 Experimental Methods

### 2.1 Materials Synthesis

Yttrium orthovanadate-phosphate powders, codoped with europium and bismuth ions, were obtained by the coprecipitation method. The concentrations of vanadium and phosphorus were fixed to 50 mol % each. Two series of materials were obtained: one in a function of bismuth concentration with fixed concentration of europium and vice versa. First, the concentration of europium ions was set to 1 mol %, while bismuth ion concentration changed from 0, 1, 3, 5, 10 up to 15 mol %. In the second series, the concentration of bismuth ions was set to 10 mol %, with concentrations of europium ion varying from 0.5, 1, 2, up to 5 mol %. Stoichiometric amounts of analytical grade Y_2_O_3_ (Alfa Aesar, 99.99%), Bi_2_O_3_ (Sigma-Aldrich, 99.9%), Eu_2_O_3_ (Alfa Aesar, 99.99%), (NH_4_)_2_HPO_4_ (ACROS Organics, >98%) and NH_4_VO_3_ (Sigma-Aldrich, 99.5%) were used in this synthesis process.

The lanthanide and bismuth oxides were converted into nitrate salts through digestion with an excess of 65% HNO_3_. Thereafter, the formed lanthanide and bismuth nitrates were recrystallized, and the HNO_3_ excess was removed. Using deionized water as a solvent, separate aqueous solutions of diammonium phosphate and ammonium metavanadate were made. The vanadium and phosphorus ion sources (NH_4_VO_3_ and (NH_4_)_2_HPO_4_) were mixed, followed by the nitrates (Y(NO_3_)_3_, Bi(NO_3_)_3_, Eu(NO_3_)_3_). The liquid mixture was stirred for 1.5 h at approximately 70 °C. Aqueous ammonia was used to maintain a pH of 9 during the reaction. The as-prepared precipitates were then washed and centrifuged at least three times, until neutral pH was reached. They were then dried for 24 h at 70 °C. The powders were finally crystallized by heat-treatment at 800 °C for 3 h in air.

### 2.2 ICP, XRD, SEM, and TEM Analyses

The crystal structure of synthesized materials was characterized by the X-ray Diffraction (XRD) technique using an X’Pert PRO X-ray diffractometer (Cu Kα1, 1.54060 Å) (PANalytical). Measured XRD patterns were compared to standards of YVO_4_ (no. 78074) and YPO_4_ (no. 79754) found in the Inorganic Crystal Structure Database (ICSD). Microstructural analyses (particle size, morphology) were performed by electron microscopy. SEM was carried out using an FEI Nova NanoSEM 230. High resolution transmission electron microscopy (HR-TEM) was performed using a Philips CM-20 Super Twin microscope. ICP-OES measurements were conducted on Thermo Scientific ICAP 7000 SERIES.

### 2.3 Spectroscopic Analysis

The Nicolet iS50 FT-IR from Thermo Scientific was used to collect infrared spectra at 300 K of samples processed in KBr pellets. The room-temperature emission spectra utilized excitation at 397, 340, and 300 nm. These spectra were collected using a FLS1000 photoluminescence spectrometer from Edinburgh Instruments. The same apparatus was used to collect the excitation spectra. The ^5^D_0_ → ^7^F_2_ transition at 619 nm was monitored at room temperature for the excitation spectra measurements. Emission spectra were also recorded in response to the 266 nm excitation of a laser diode (CW) at room temperature and detected using the Hamamatsu PMA-12 photonic multichannel analyzer. The emission decay profiles were measured at 300 K using either a Ti:sapphire tunable laser or a Nd:YAG laser, a Hamamatsu R928 photomultiplier, a Jobin-Yvon THR 1000 spectrophotometer, and a digital LeCroy WaveSurfer oscilloscope. Excitation and emission spectra were collected at low temperature (5 K) using a temperature-controlled, continuous-flow liquid helium cryostat: Oxford Model CF 1204. Low temperature excitation spectra were measured with a Dongwoo Optron DM151i monochromator and a 150W ozone free lamp. The low temperature emission spectra were measured using a Dongwoo Optron DM750 monochromator, an Electro-Optical System INC PbS photodiode, or a Hamamatsu R928 photomultiplier.

## 3 Results and Discussion

### 3.1 Structure and Morphology

The chemical composition of all samples, analyzed by ICP-OES, is given in Table 1. It is verified that the nominal compositions are congruous with actual compositions.

**Table 1:** Elemental Composition of the Europium and Bismuth Codoped YV_0.5_P_0.5_O_4_ in Molar Percentages

	YV_0.5_P_0.5_O_4_								
element	0.5% Eu^3+^ 10% Bi^3+^	1% Eu^3+^ 10% Bi^3+^	2% Eu^3+^ 10% Bi^3+^	5% Eu^3+^ 10% Bi^3+^	1% Eu^3+^ 1% Bi^3+^	1% Eu^3+^ 3% Bi^3+^	1% Eu^3+^ 5% Bi^3+^	1% Eu^3+^ 10% Bi^3+^	1% Eu^3+^ 15% Bi^3+^
Y	89.08	98.73	87.83	84.97	97.95	95.47	92.42	88.91	83.66
V	49.70	49.63	50.05	49.65	50.32	50.55	49.79	49.78	50.06
P	49.79	49.54	49.89	50.27	50.54	48.73	49.84	49.47	49.74
Eu	0.52	1.01	1.95	4.98	1.03	1.03	1.03	1.02	1.05
Bi	10.06		10.01	10.10	0.96	3.03	4.97	9.98	14.94

**Figure 2:**
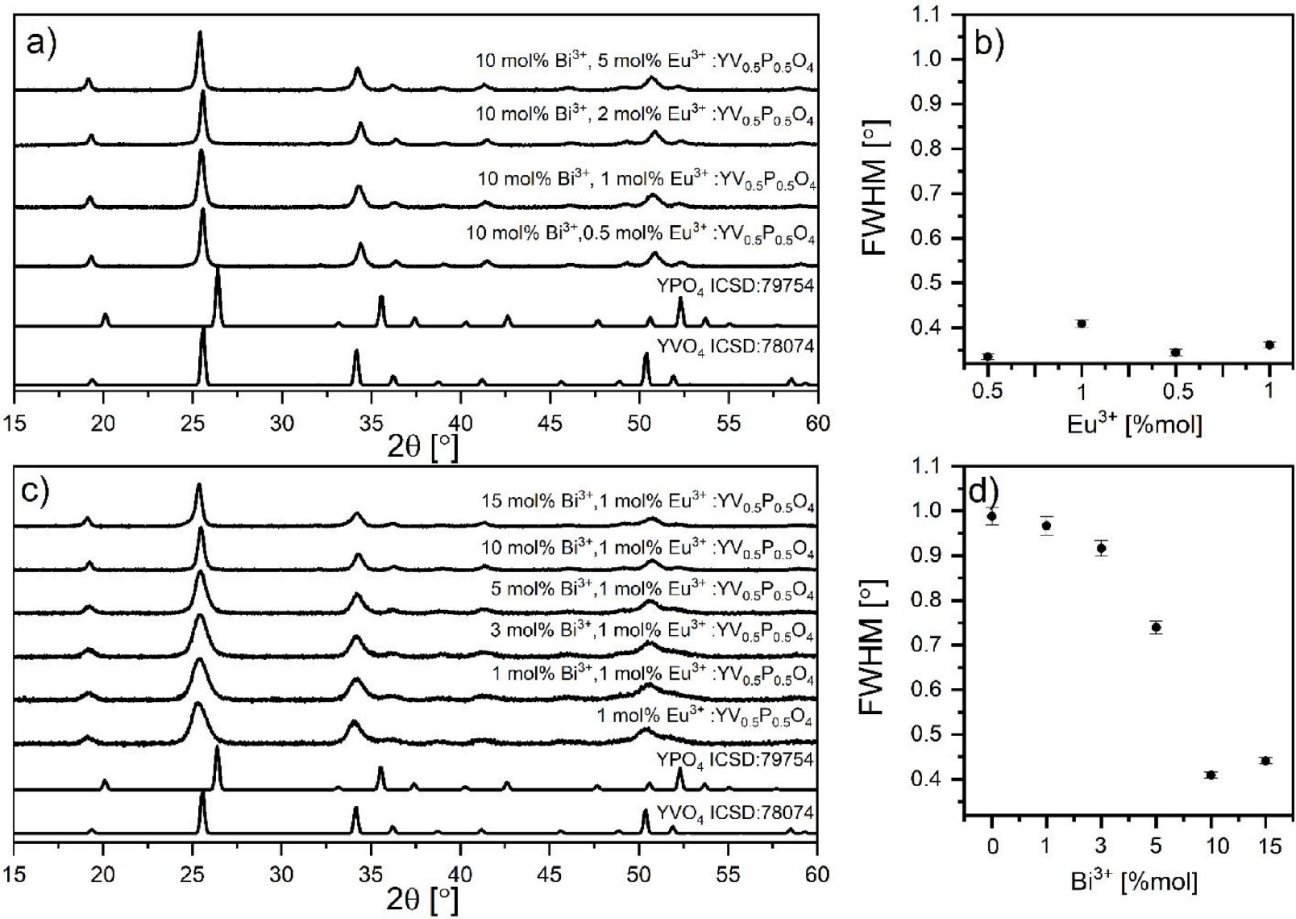
Diffractograms (a, c) and fwhm analysis (b, d) obtained for *x* mol % Bi^3+^, 1 mol % Eu^3+^: YV_0.5_P_0.5_O_4_ and 10 mol % Bi^3+^, *y* mol % Eu^3+^: YV_0.5_P_0.5_O_4_.

XRD results confirmed the crystal phase purity of YV_0.5_P_0.5_O_4_ doped derivatives (Figure 2). The XRD peaks are broadened and further confirm the structural disorder, as observed earlier.^10,12,20^ This broadening originates from the size difference between V^5+^ (0.36 Å, C.N. 4) and P^5+^ (0.17 Å, C.N. 4), when statistically distributed in YV_*x*_P_1–*x*_O_4_ solid solution. The lattice strains are influenced by changes in grain size. This is a result of point defects, vacancies,^34^ and varying composition^35^ as well as dislocations near the grain-boundaries^36^ caused by the incompatibility of phosphorus and vanadium atoms. The observed changes in the fwhm of the XRD peaks may indicate the presence of lattice strains.^37^

Co-doping with Eu^3+^ does not impact the lattice and, by extension, the width of the XRD peaks. In contrast, it is found that codoping with Bi^3+^ contributes to narrowing of the XRD peaks (Figure 2c). This is clearly evidenced by Figure 2b,d. Additionally, increasing the Eu^3+^ amount in 10 mol % Bi^3+^ codoped samples does not affect the XRD peak widths significantly.

**Figure 3:**
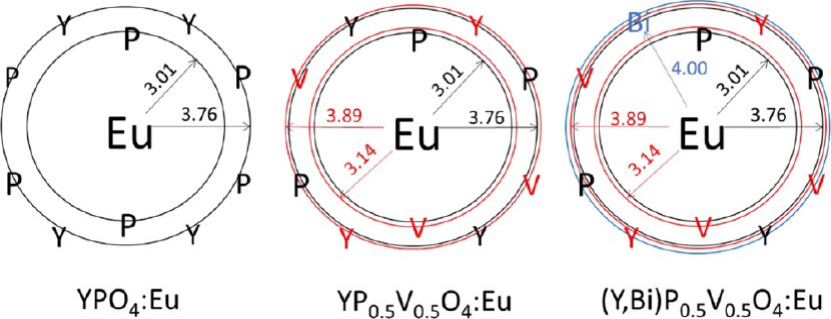
Cation coordination spheres of Eu^3+^ in zircon-like YPO_4_, YP_0.5_V_0.5_O_4_, and (Y,Bi)P_0.5_V_0.5_O_4_. Oxygen atoms are not represented.

By considering Eu^3+^ as a local luminescent structural probe, we find three possible environments experienced by Y^3+^ ions in the compounds. This is depicted in Figure 3. In YPO_4_, the Eu^3+^ ions are surrounded by eight O atoms, thus forming a dodecahedron with *D*_2*d*_ point symmetry. The first cation coordination consists of two P^5+^ at 3.01 Å and respectively four P^5+^ and four Y^3+^ at 3.76 Å in a second coordination. The exact charge (the BVS - Bond Valence Sum) carried by these cations is given in Figure 1. Incorporation of 50% V^5+^ creates two additional spheres at 3.14 and 3.89 Å (marked as red in Figure 3) with statistical occupancy. Incorporation of Bi^3+^ results in a third sphere (marked as blue in Figure 3) at 4.00 Å. The probability of finding a Bi^3+^ ion in this position is P(*x*) = 1–(1–*x*)^4^ wherein *x* is the molar percentage of Bi^3+^, e.g., P(0.1) = 35%. The bismuth ions (1.17 Å at C.N. 8) substitute yttrium ions (1.02 Å at C.N. 8) in a statistical manner. Since Bi^3+^ is about 15% larger than Y^3+^, its incorporation in the crystal lattice counterbalances (at least partly) the ionic radius mismatch between V^5+^ and P^5+^. This inverse relationship between V^5+^–P^5+^ and Bi^3+^–Y^3+^ ionic radii mismatches reduces the lattice strains in YV_0.5_P_0.5_O_4_ and thereby sharpens the XRD lines as the Bi^3+^ concentration is raised (Figure 2c).

**Figure 4:**
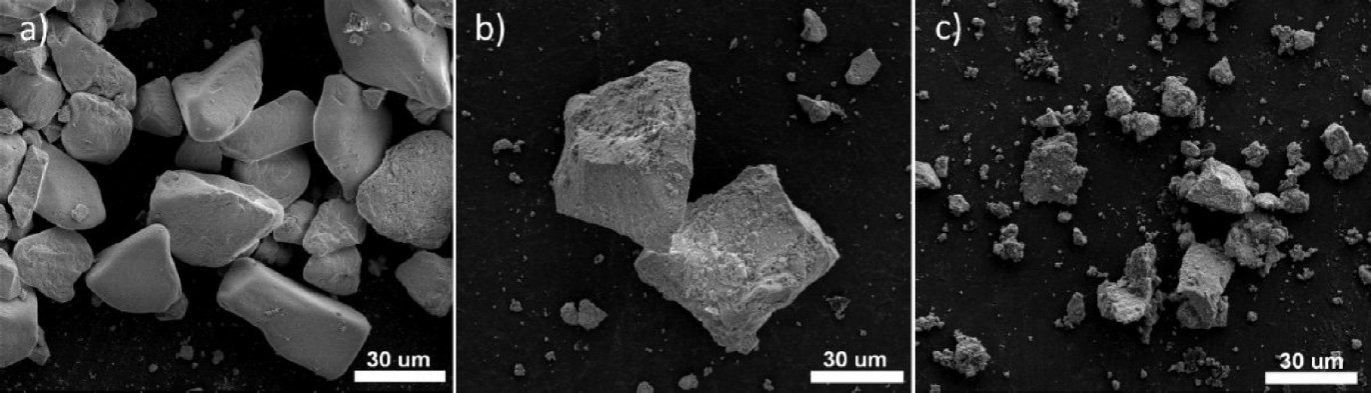
SEM images obtained for YV_0.5_P_0.5_O_4_: 1 mol % Eu^3+^, *x*Bi^3+^, where *x* = 5 mol % a), 10 mol % b), and 15 mol % c).

**Figure 5:**
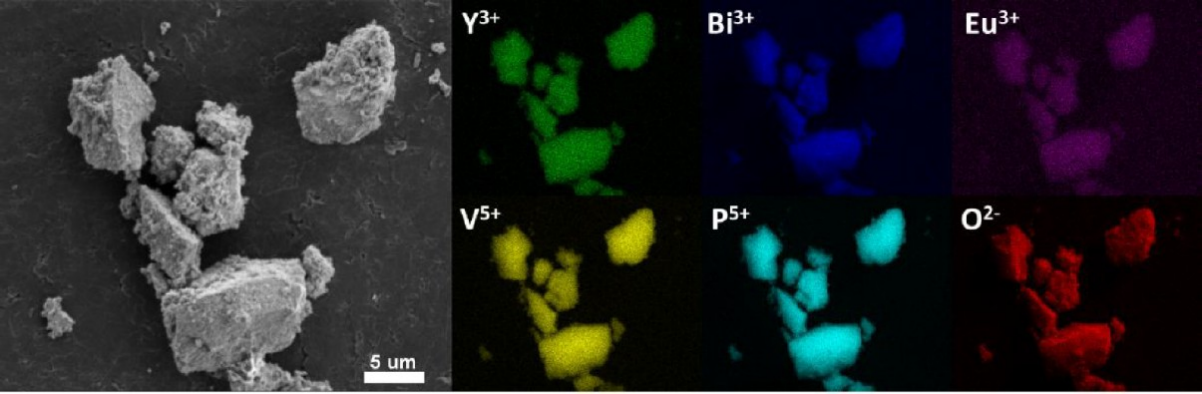
EDS maps obtained for 1 mol % Eu^3+^, 10 mol % Bi^3+^: YV_0.5_P_0.5_O_4_.

SEM images of YV_0.5_P_0.5_O_4_: 1 mol % Eu^3+^, *x* mol % Bi^3+^ materials are depicted in Figure 4. The powders look micrometric regardless of bismuth ion concentration. As the bismuth content is raised, the particles become smaller, and their surfaces become rougher. EDS maps (Figure 5) obtained for 1 mol % Eu^3+^, 10 mol % Bi^3+^: YV_0.5_P_0.5_O_4_ confirm random distribution of the constituents.

**Figure 6:**
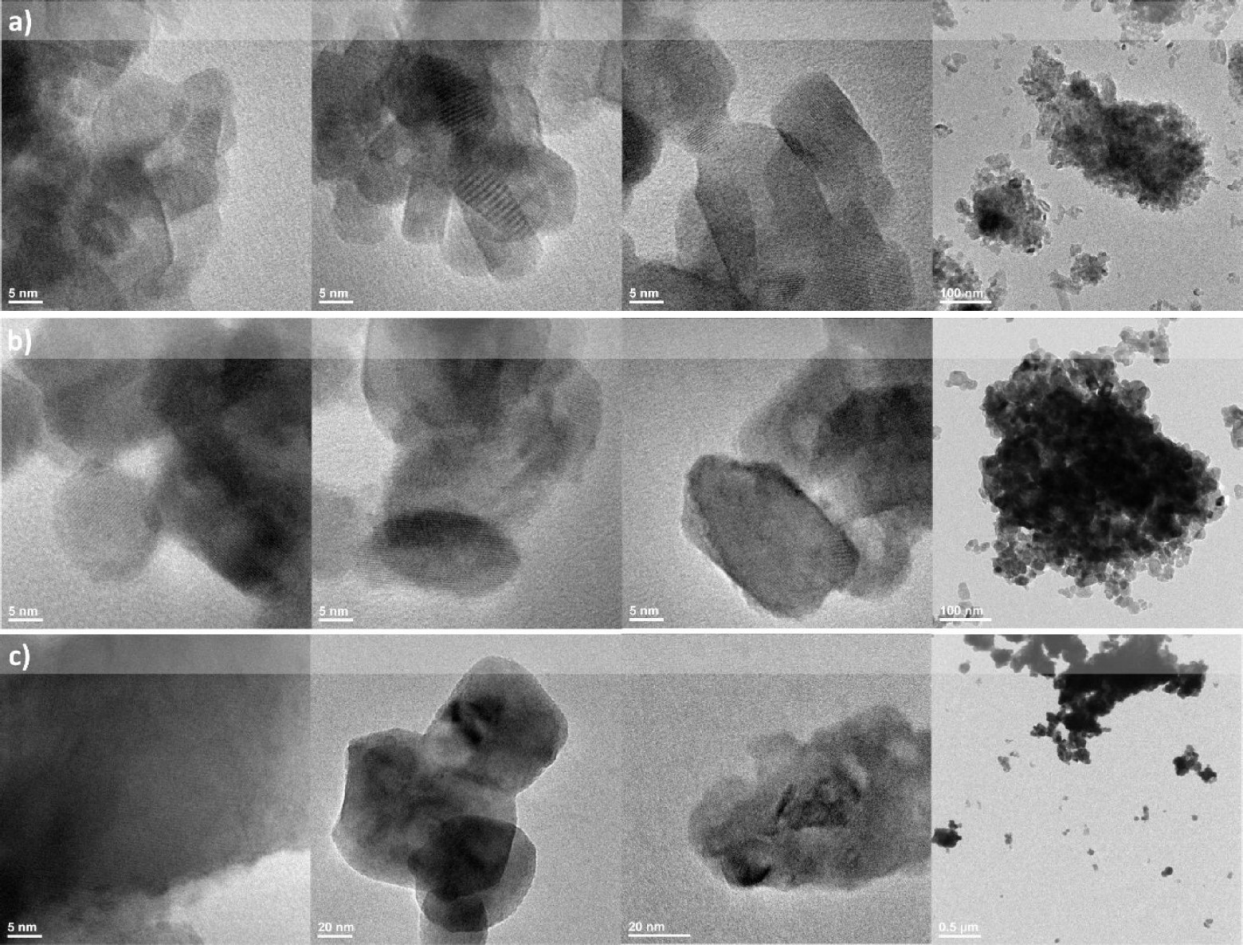
TEM images of YV_0.5_P_0.5_O_4_ doped with 1 mol % Eu^3+^ and 1 mol % Bi^3+^ a), 1 mol % Eu^3+^ and 5 mol % Bi^3+^ b), and 1 mol % Eu^3+^ and 15 mol % Bi^3+^ (c).

The TEM images in Figure 6 demonstrate that the aggregates consist in fact of nanosized particles. Analysis of the SEM (Figure 5) and TEM (Figure 6) images reveals a wide distribution of particle sizes among the samples, although we note that the particles size increases as the Bi^3+^ content is raised (i.e., 19, 31, and 62 nm for doping rates of 1, 5, and 15 mol %, respectively). Differences in particles sizes contribute to the narrowing of the XRD peaks in addition to the reordering of the crystal structure. This work sheds a light into a complex structure of Eu^3+^, Bi^3+^: YV_0.5_P_0.5_O_4_. However, to individuate or quantify the role of each effect, further crystallographic research needs to be conducted. Additionally, *d*-spacing values were calculated by the means of FFT processing in ImageJ software. d-Spacing was calculated to be *d*_101_ = 0.45 nm for YV_0.5_P_0.5_O_4_ samples codoped with 1 mol % Eu^3+^ and 1 mol % Bi^3+^ and 1 mol % Eu^3+^ and 5 mol % Bi^3+^, as well as 1 mol % Eu^3+^ and 15 mol % Bi^3+^. Also *d*_200_ values were calculated. For samples codoped with 1 mol % Eu^3+^ and 1 mol % Bi^3+^, *d*_200_ was 0.35 nm. However, 5 mol % and 15 mol % Bi^3+^-doped samples had a *d*_200_ value of 0.33 nm. All *d*-spacing values coincide with *d*_101_ and *d*_200_ values from standard patterns of YVO_4_ (*d*_101_ = 0.47132 nm, *d*_200_ = 0.35591 nm) and YPO_4_ (*d*_101_ = 0.45379 nm, *d*_200_ = 0.34474 nm).

### 3.2 Spectroscopic Properties

**Figure 7:**
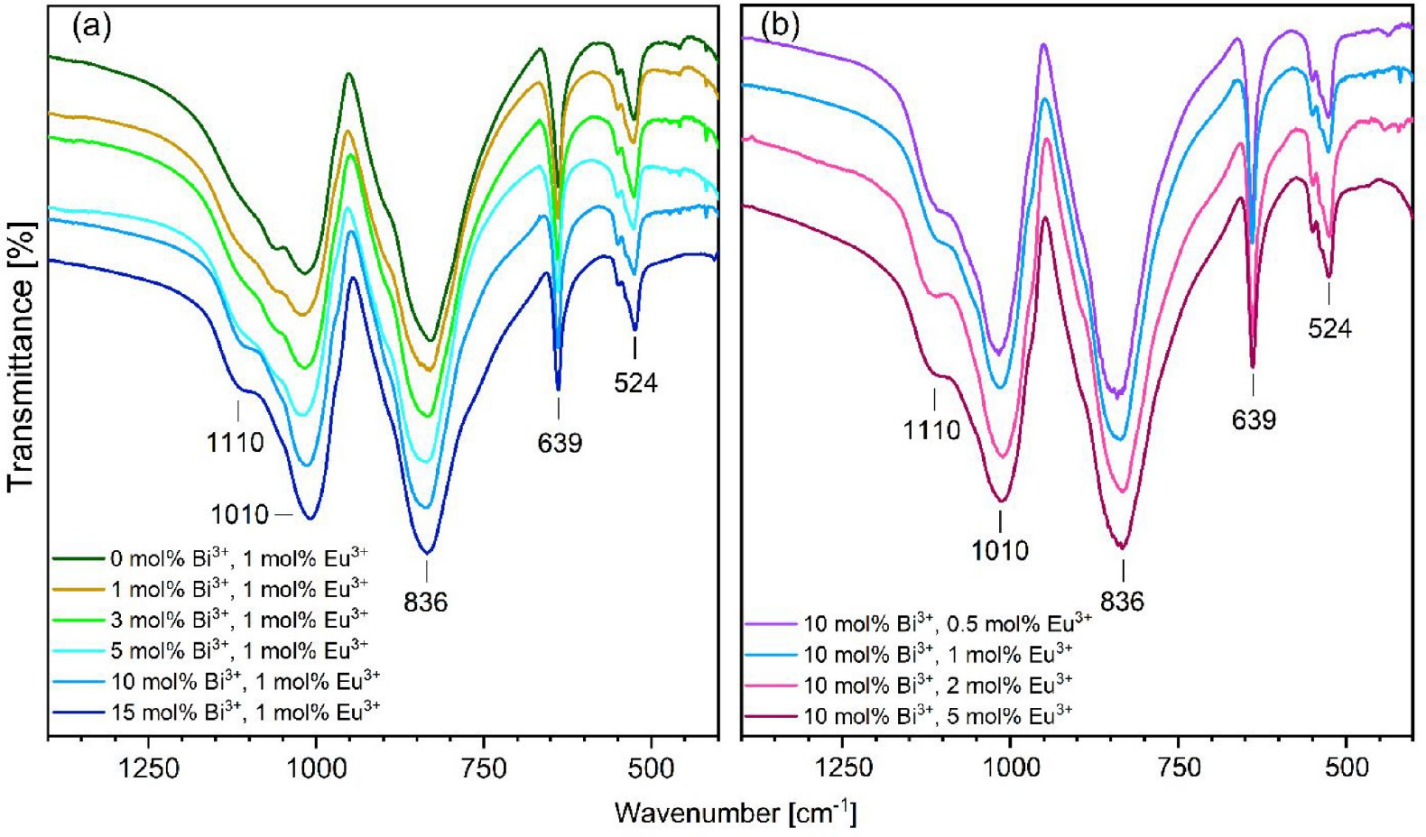
Fourier transformed infrared spectra of (a) codoped with Bi^3+^ and 1 mol % Eu^3+^ YV_0.5_P_0.5_O_4_, with varying Bi^3+^ concentrations and (b) codoped with 10 mol % Bi^3+^ and Eu^3+^, with varying Eu^3+^ concentrations.

The FT-IR spectra of YV_0.5_P_0.5_O_4_ codoped with Eu^3+^ and Bi^3+^ are shown in Figure 7. There are five strong absorption bands in the range of 1300–400 cm^–1^. The peaks at 524 cm^–1^ and at 639 cm^–1^ represent an antisymmetric bending vibration of ν_4_(PO_4_)^3–^. The antisymmetric stretching vibration of ν_3_(PO_4_)^3–^ can be found at 1010 cm^–1^ and at 1110 cm^–1^.^38,39^ The peak at 836 cm^–1^ is ascribed to the vibration mode of the (VO_3_)^−^ group. The weak peak detected at 502 cm^–1^ is related to the Y–O vibration.^40^ This mode is not observed for the samples with more than 10 mol % of codopant ions concentration. The Bi–O modes are on the verge of our measurement range.^41,42^

**Figure 8:**
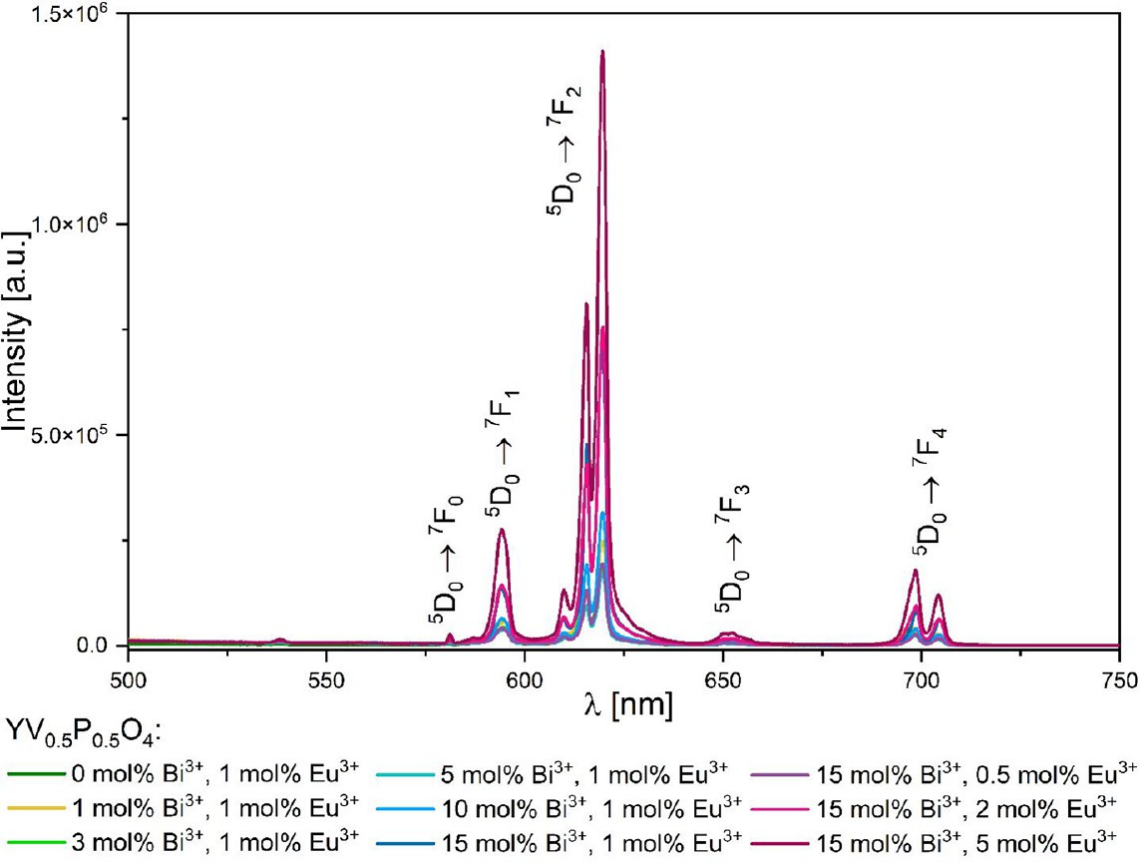
Emission spectra for *x* mol % Bi^3+^, *y* mol % Eu^3+^: YV_0.5_P_0.5_O_4_ under 397 nm excitation at room temperature.

Room temperature measurements revealed that all compounds exhibit red emission typical of Eu^3+^ upon direct *4f-4f* excitation at 397 nm (Figure 8). This emission increases in intensity with increasing Eu^3+^ and Bi^3+^ concentrations. The highest observed emission intensity is in samples containing 10 mol % Bi^3+^, 5 mol % Eu^3+^: YV_0.5_P_0.5_O_4_.

**Figure 9:**
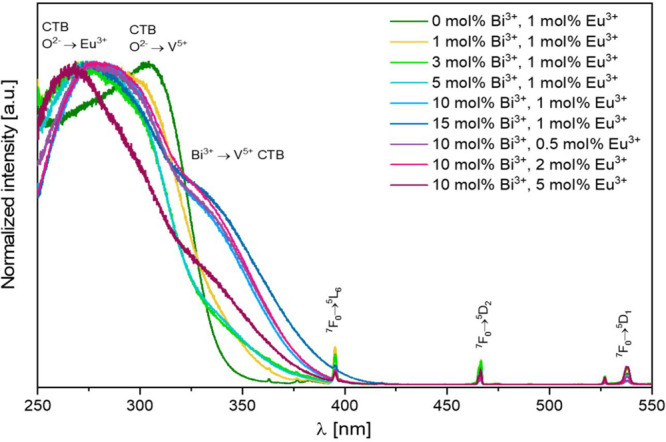
Excitation spectra for *x* mol % Bi^3+^, *y* mol % Eu^3+^: YV_0.5_P_0.5_O_4_ materials measuring the intensity of the ^5^D_0_ → ^7^F_2_ transition at 619 nm at room temperature.

The excitation spectra for *x* mol % Bi^3+^, *y* mol % Eu^3+^: YV_0.5_P_0.5_O_4_ materials for the ^5^D_0_ → ^7^F_2_ transition at 619 nm are shown in Figure 9. Three broad transitions are observed at 266, 300, and ≈340 nm (shoulder). They correspond to the O^2–^ → Eu^3+^, O^2–^ → V^5+^, and Bi^3+^ → V^5+^ charge transfers, respectively.^2,13,14,33^ The intrinsic *4f-4f* excitation lines of Eu^3+^ (namely ^7^F_0_ → ^5^L_6_, the ^7^F_0_ → ^5^D_2_, and ^7^F_0_ → ^5^D_1_ transitions) are comparatively much less intense.

**Figure 10:**
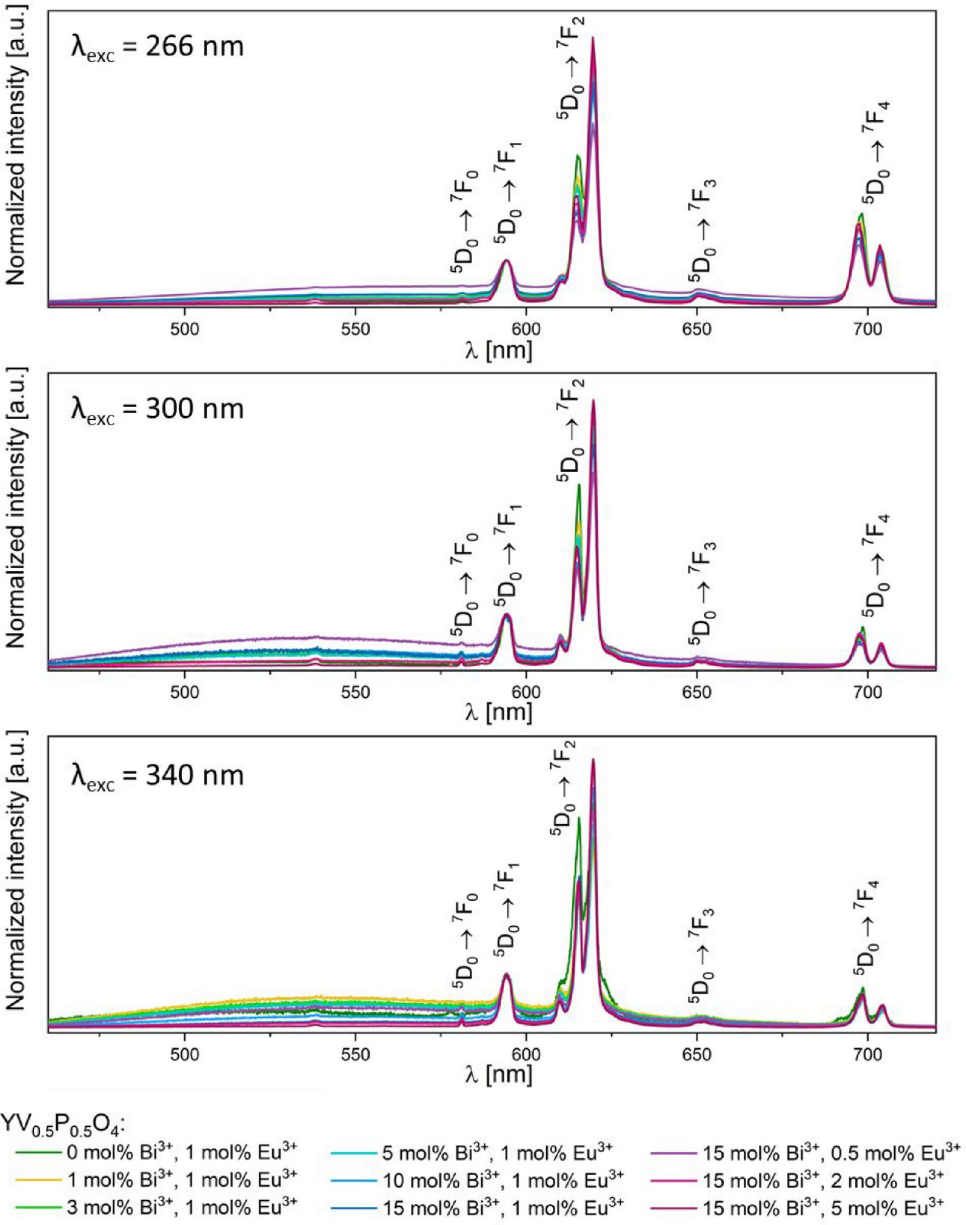
Emission spectra for *x* mol % Bi^3+^, *y* mol % Eu^3+^: YV_0.5_P_0.5_O_4_ under 266, 300, and 340 nm excitation normalized to the ^5^D_0_ → ^7^F_1_ transition of Eu^3+^.

Figure 10 depicts the emission spectra after excitation in the charge transfer bands. These intensities are normalized to the ^5^D_0_ → ^7^F_1_ transition of Eu^3+^. In addition to characteristic emission lines of Eu^3+^, broad emission signals are observed. Upon 340 nm excitation, the broad signal represents the emission of the Bi–V metal-to-metal CT,^15^ whereas upon 300 nm excitation, the broad signal is more surely due to perturbed vanadate groups. Upon 266 nm excitation, these bandlike emissions possibly overlap. The presence of these emission bands indicates an incomplete sensitization of Eu^3+^ luminescence. Two possible sensitization paths are identified. They involve the (VO_4_)^3-^ units or the Bi–V self-trapped excitons as energy donors and the Eu^3+^ ions as energy acceptors. Decay profiles were collected to quantify the efficiency of these energy transfers.

**Figure 11:**
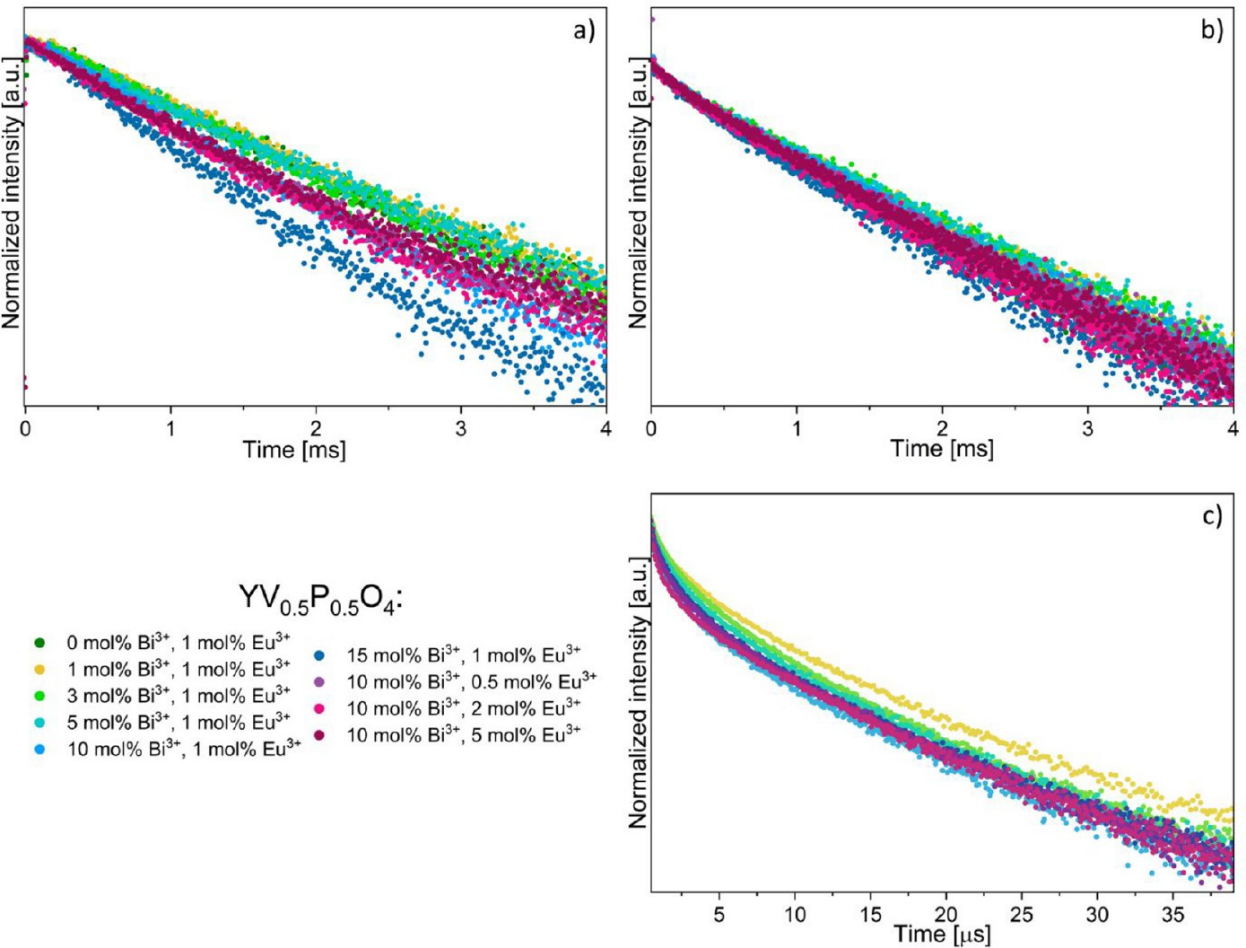
Decay time profiles measured for ^5^D_0_ → ^7^F_2_ transition monitored at 619 nm λ_exc_ = 397 nm (a), ^5^D_0_ → ^7^F_2_ transition, λ_em_ = 619 nm and λ_exc_ = 355 nm (b), and decay profiles measured for MMCT-Bi transition λ_em_ = 540 nm and λ_exc_ = 355 nm (c).

**Figure 12:**
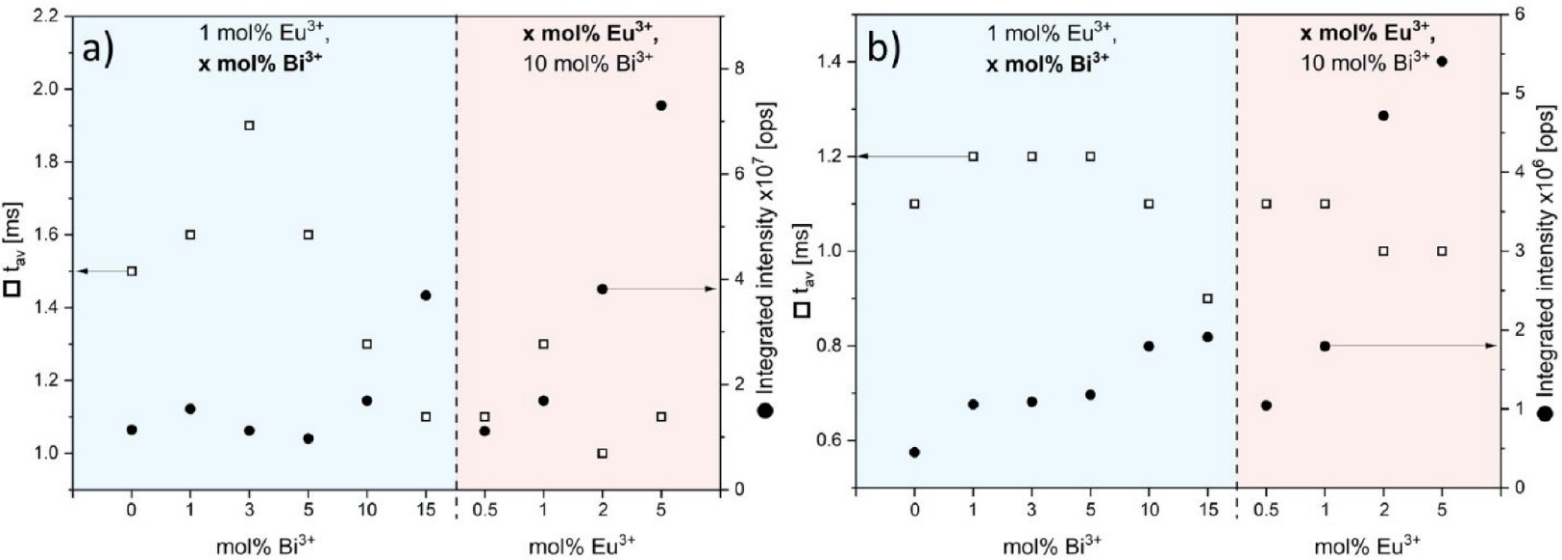
Integrated ^5^D_0_ → ^7^F_2_ emission intensity (●) and average emission lifetime of Eu^3+^ (□) for the different compounds listed in Table 2 in correspondence with λ_exc_ = 397 nm (a) and λ_exc_ = 355 nm (b).

Decay profiles are presented in Figure 11 for 397 nm excitation (inner ^7^F_0_ → ^5^L_6_ Eu^3+^ transition) or 355 nm excitation (Bi–V MMCT). Corresponding average values of the luminescence lifetimes *t*_av_, were calculated as *t*_av_ = ∫*I*(*t*)*t* d*t*/(∫*I*(*t*) d*t*). In this case, *I*(*t*) represents the emission intensity at time *t*. These values are provided in Table 2. A plot is proposed in Figure 12 for discussion.

**Table 2:** Calculated Average Decay (*t*_av_) and Rise (*t*_rise_) Times for *x* mol % Bi^3+^, *y* mol % Eu^3+^: YV_0.5_P_0.5_O_4_ Pumped with 397 and 355 nm Wavelengths^a^

		^5^D_0_ → ^7^F_2_ (Eu^3+^) transition at 619 nm		Bi–V MMCT transition at 540 nm	
		λ_exc_ = 397 nm	λ_exc_ = 355 nm	λ_exc_ = 355 nm	
label	YV_0.5_P_0.5_O_4_	*t*_av_ [ms]	*t*_av_ [ms]	*t*_av_ [μs]	η (%)
A	1 mol % Eu^3+^	1.5	1.1		
B	1 mol % Bi^3+^			7.0	
C	1 mol % Bi^3+^, 1 mol % Eu^3+^	1.6	1.2	6.9	1.1
D	3 mol % Bi^3+^, 1 mol % Eu^3+^	1.9	1.2	5.8	16.5
E	5 mol % Bi^3+^, 1 mol % Eu^3+^	1.6	1.2	5.8	17.2
F	10 mol % Bi^3+^, 1 mol % Eu^3+^	1.3	1.1	5.5	20.7
G	15 mol % Bi^3+^, 1 mol % Eu^3+^	1.1	0.9	5.6	19.5
H	10 mol % Bi^3+^, 0.5 mol % Eu^3+^	1.1	1.1	5.7	17.9
I	10 mol % Bi^3+^, 2 mol % Eu^3+^	1.0	1.0	5.9	16.2
J	10 mol % Bi^3+^, 5 mol % Eu^3+^	1.1	1.0	6.1	12.9

a Each sample composition was assigned a label to facilitate the discussion below.

The parameter η in Table 2 is related to the efficiency of the ET from Bi^3+^-to-Eu^3+^ in the codoped compounds. It is obtained from the equation
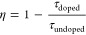
where τ_doped_ is the time constant in the presence of the Eu^3+^ acceptor, and τ_undoped_ is the time constant without Eu^3+^ (i.e., only Bi^3+^).

Figure 12(a) pertains to the inner ^7^F_0_ → ^5^L_6_ excitation of Eu^3+^. The left-hand side of the vertical dashed line corresponds to 1 mol % Eu^3+^ doped samples with increasing Bi^3+^ concentrations (compound A to compound H, excluding compound B, in Table 2). Here, an increase in emission lifetime with increasing bismuth concentration is observed up to 3 mol % Bi^3+^ (compound D). These samples (A,C,D,E) also exhibit lower emission intensity relative to samples with higher Bi^3+^ concentrations. This suggests a lower radiative probability from the ^5^D_0_ state in these cases. Raising the Bi^3+^ amount further contributes to enhance the Eu^3+^ emission intensity, and it correlates with a shortening of the lifetime. This effect has already been observed in YPO_4_:Sm^3+^, Bi^3+^ and is ascribed to an increased refractive index of the host lattice.^43^ In this case, the radiative probability is increased, contributing to a lower emission lifetime. A synergetic effect with the reordering of the crystal structure is not excluded. This requires further investigation. The right-hand side of the figure shows the effect of Eu^3+^ content for a fixed amount of Bi^3+^. In this case, the emission lifetime does not vary significantly because the medium’s refractive index is unchanged. Here, the increase in emission intensity is ascribed to the larger Eu^3+^ content that remains beyond the quenching concentration.^44,45^

Part (b) of Figure 12 relates to an excitation in the Bi–V MMCT band, the intensity of which exceeds by far that of the ^7^F_0_ → ^5^L_6_ transition (Figure 9). In this situation, the Eu^3+^ emission is produced after an energy transfer whose efficacy is given in Table 2. Efficacy is 20% in the Bi^3+^-rich compounds F and G but tends to fall off in compounds containing more than 1% Eu^3+^: η is for instance comparable in compound D (3 mol % Bi^3+^, 1 mol % Eu^3+^) and in compound I (10 mol % Bi^3+^, 2 mol % Eu^3+^). Nevertheless, the emission intensity of compound J amounts to 6.5 times that of compound D, which demonstrates a synergy between Bi^3+^ and Eu^3+^ contents in YV_0.5_P_0.5_O_4_. Furthermore, with respect to both 355 and 397 nm excitations, compound J exhibits the highest emission intensity related to direct Eu^3+^ excitation.

At sufficiently low temperatures, vibronic interactions can be frozen out. Thus, the luminescence spectra can give more detailed information regarding the electronic transitions of Eu^3+^ and can now be used as a structural probe. In 2008, Pan et. al^46^ conducted investigations regarding the spectroscopic properties of Eu^3+^ in Y(V,P*)*O_4_ solid solution by laser-selective excitation. This work identified three symmetry sites in the yttrium orthovanadate-phosphate mixed compounds due to disorder generated by the distribution of (PO_4_) and (VO_4_) tetrahedra. The Judd–Ofelt intensity parameters further confirmed that significant changes in ligand polarizability contribute to differences in local environments experienced by Eu^3+^.^46^

**Figure 13:**
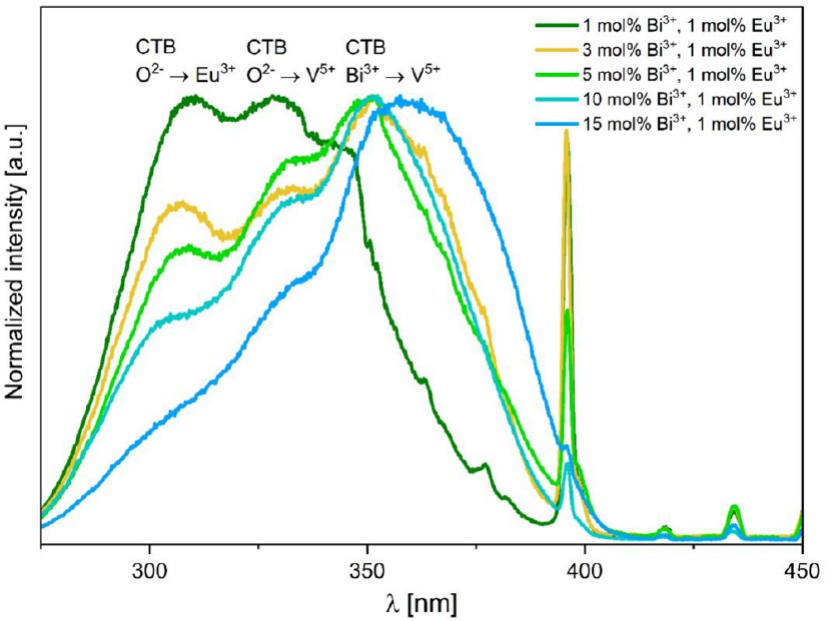
Excitation spectra for *x* mol % Bi^3+^, 1 mol % Eu^3+^: YV_0.5_P_0.5_O_4_ materials monitoring the 619 nm emission corresponding to the ^5^D_0_ → ^7^F_2_ transition at *T* = 5 K.

Figure 13 illustrates the excitation spectra collected at 5 K corresponding to the 619 nm emission which represents the Eu^3+^^5^D_0_ → ^7^F_2_ transition. Low temperature excitation spectra depict noticeable differences when compared to the room temperature spectra. These major differences are as follows:

(1) differences in relative intensity of the intrinsic ^7^F_0_ → ^5^L_6_ transitions of Eu^3+^ to the CT excitation bands in compounds containing low amounts of Bi^3+^ (<5 mol %). This indicates a less efficient sensitization at 5 K. Furthermore, it demonstrates that in these conditions, the Bi-to-Eu energy transfer is efficiently phonon assisted. However, even at 5 K the Bi-to-Eu energy transfer regains efficacy when the Bi^3+^ concentration is 10 mol %. This coincides with the increased presence of a broad excitation band which peaks at ≈360 nm and extends up to ≈400 nm, currently attributed to Bi–V MMCT.
(2) the presence of structures on the Bi–V MMCT excitation with maxima identified at ≈350, 330, and 307 nm. The relative intensity of these excitation maxima strongly depends on the Bi^3+^ content in the compound. There is a notable red shift in the excitation spectra as Bi^3+^ concentration increases. This shift of the Bi–V excitation edge with increasing the Bi content has been noted in previous studies, e.g., in ref^32^.
(3) the absence of excitation features pertaining to O^2–^ → Eu^3+^ and O^2–^ → V^5+^ charge transfers. This suggests these transitions are not involved in the sensitization process of Eu^3+^ at 5 K. Therefore, only the Bi–V MMCT operates as a sensitizing channel for Eu^3+^ at 5 K.

**Figure 14:**
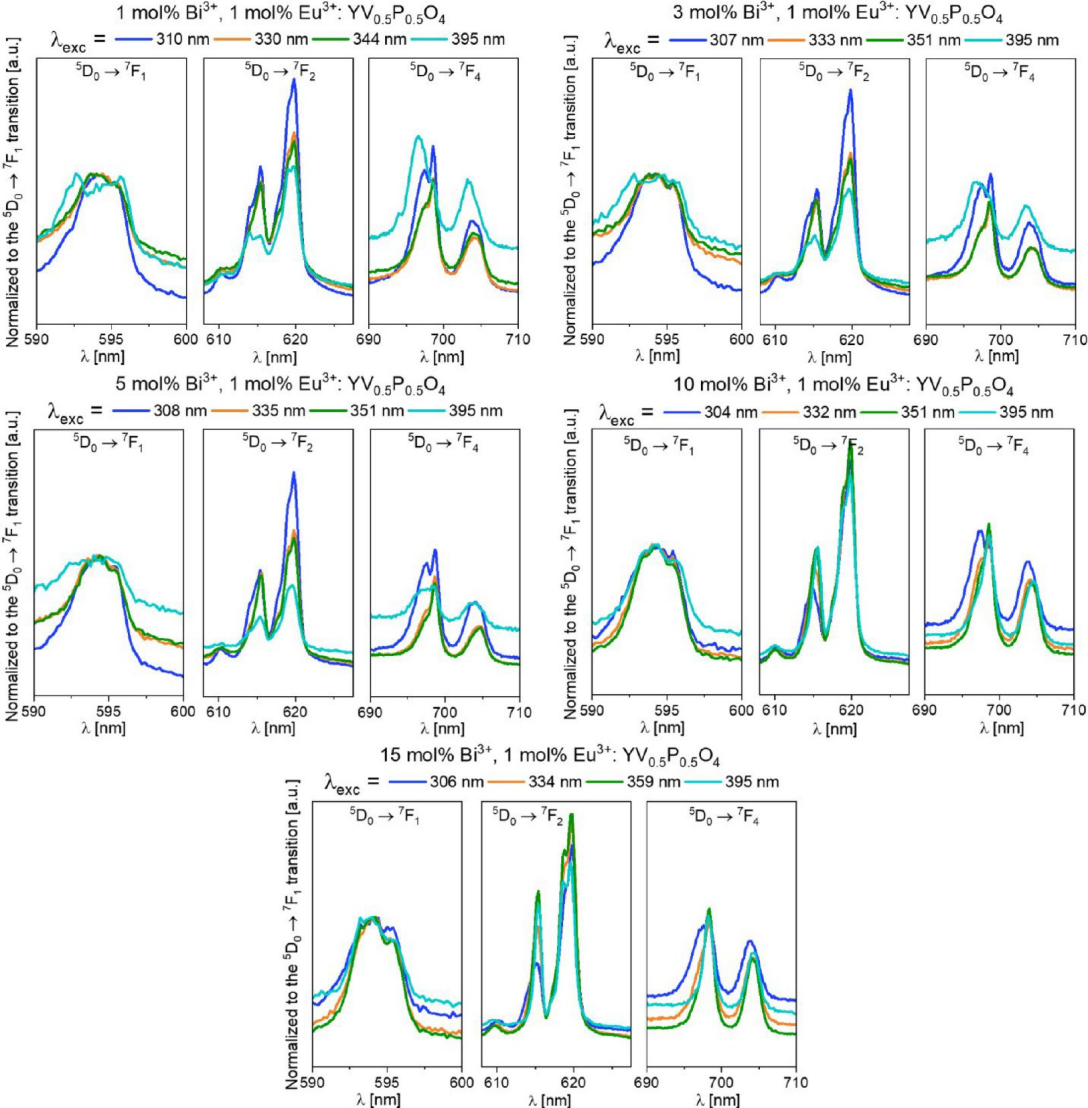
Emission spectra of 1 mol % Eu^3+^ in Y_1–*x*_Bi_*x*_V_0.5_P_0.5_O_4_ (*x* = 0.01 to 0.15) upon various excitation wavelengths at 5 K. Spectra normalized to the ^5^D_0_ → ^7^F_1_ transition of Eu^3+^ .

The emission spectra for 1, 3, 5, 10, 15 mol % Bi^3+^, 1 mol % Eu^3+^: YV_0.5_P_0.5_O_4_ were collected at 5 K. These samples were excited in the CT bands and at 395 nm (Figure 14). The wavelengths of the Stark components observed in Y_0.98_Eu_0.01_Bi_0.01_V_0.5_P_0.5_O_4_ and Y_0.89_Eu_0.01_Bi_0.10_V_0.5_P_0.5_O_4_ are compiled in Table 3 and compared to reference data on Eu^3+^ in YPO_4_ and YVO_4_.^47^

**Table 3:** Stark Components (in nm) of Eu^3+^ in YVO_4_,^47^ YPO_4_,^47^ and Y_1–*x*_Bi_*x*_V_0.5_P_0.5_O_4_ (*x* = 0.01, and 0.10)^b^

^5^D_0_ →	YPO_4_	YVO_4_	Y_0.98_Eu_0.01_Bi_0.01_V_0.5_P_0.5_O_4_	Y_0.89_Eu_0.01_Bi_0.10_V_0.5_P_0.5_O_4_
^7^F_0_	581.0	581.9		
^7^F_1_	592.7	593.5		
	596.1	595.0	594.7^a^	594.5^a^
^7^F_2_	613.4	615.5	610.5	609.9
	617.6	617.3	613.7	615.4
	619.3	619.4	615.3	618.8
	620.2	622.4	619.0	619.9
			619.9	
^7^F_4_	691.6	690.5	696.7	698.4
	694.4	696.7	698.6	704.0
	696.2	698.5	703.4	
	697.3	699.1		
	699.3	701.2		
	703.5	704.5		
	704.7	708.2		

a Centroid position.

b Data were taken at 5 K.

Data related to the ^5^D_0_ → ^7^F_4_ transition of Eu^3+^ are incomplete, when compared to the reference zircon compounds YPO_4_:Eu^3+^ and YVO_4_:Eu^3+^. The ^5^D_0_ → ^7^F_1_ transitions are also poorly resolved despite the low temperature. Thereby the results are discussed based on the ^5^D_0_ → ^7^F_2_ transitions. Two different signatures are noted for the Eu^3+^ transitions in Y_0.98_Eu_0.01_Bi_0.01_V_0.5_P_0.5_O_4_ depending on the excitation wavelength. This indicates the presence of more than one Eu^3+^ type site in this crystal structure, which agrees with the conclusions of Pan et al.^46^ In Y_0.89_Eu_0.01_Bi_0.10_V_0.5_P_0.5_O_4_, however, only four Stark components (instead of five) corresponding to the ^5^D_0_ → ^7^F_2_ transition were observed. The spectrum in the ^5^D_0_ → ^7^F_4_ region looks also simpler. This simplification of the spectral signatures is consistent with the progressive crystal structure reordering of YP_0.5_V_0.5_O_4_ concomitant with an increase in Bi^3+^ concentration. This is previously observed in XRD results (Figure 2). Compared to YPO_4_:Eu^3+^ and YVO_4_:Eu^3+^, the ^5^D_0_ → ^7^F_2_ spectrum of Y_0.89_Eu_0.01_Bi_0.10_V_0.5_P_0.5_O_4_ is more split (265 cm^–1^ against 178–180 cm^–1^), and its energy barycenter is upshifted. This is due to the presence of the Bi^3+^ ion in the second cationic neighborhood of Eu^3+^, as we have depicted in Figure 3. Owing to the BVS values given in Figure 1, it is concluded that the formal charge carried by bismuth is below that of yttrium, with the consequence that the formal charge carried by phosphorus and vanadium atoms in the Bi-doped compounds is enhanced with respect to the Bi-free compounds. This, in turn, affects the formal charge carried by the oxygen atoms in the first coordination of Eu^3+^ by reinforcing the crystal field and softening the nephelauxetic effect.

## 4 Conclusion

The pure crystal phase of *x* mol % Bi^3+^, *y* mol % Eu^3+^: YV_0.5_P_0.5_O_4_ was formed using a coprecipitation synthesis method. It is observed that codoping with Bi^3+^ is followed by narrowing of the XRD peaks, but codoping with Eu^3+^ does not significantly affect the widths of the peaks. The insufficient energy transfer to Eu^3+^, resulting from the Bi–V MMCT and the ^3^T_1,2_ → ^1^A_1_ (VO_4_)^3–^ broad transition bands, is observed. Yet, Eu^3+^ ion emission is enhanced by increasing Bi^3+^ ion concentration. Further reaffirmed is the presence of more than one Eu^3+^ site due to (PO_4_)^3–^ and (VO_4_)^3–^ substitution. Additionally, an ordering of the crystal structure of YP_0.5_V_0.5_O_4_ with increasing the Bi^3+^ content can be observed. It is supposed that at low temperature the sensitizing pathway of Eu^3+^ is less efficient in comparison to the room temperature, and mainly the Bi–V MMCT contribution is noticeable. Based on luminescent decay times, the Bi–Eu ET efficiency was found to be the highest for bismuth-rich compounds.
